# Development and clinical validation of inertial sensor-based gait-clustering methods in Parkinson’s disease

**DOI:** 10.1186/s12984-019-0548-2

**Published:** 2019-06-26

**Authors:** An Nguyen, Nils Roth, Nooshin Haji Ghassemi, Julius Hannink, Thomas Seel, Jochen Klucken, Heiko Gassner, Bjoern M. Eskofier

**Affiliations:** 1 Machine Learning and Data Analytics Lab, Department of Computer Science, Friedrich-Alexander-University Erlangen-Nürnberg (FAU), Carl-Thiersch-Straße 2b, Erlangen, 91052 Germany; 2 Department of Molecular Neurology, University Hospital Erlangen, Friedrich-Alexander University Erlangen-Nürnberg (FAU), Schwabachanlage 6, Erlangen, 91054 Germany; 3 Control Systems Group, Department of Electrical Engineering and Computer Science, Technische Universität Berlin (TUB), Einsteinufer 17, Berlin, 10587 Germany

**Keywords:** Parkinson’s disease, Gait analysis, Inertial sensors, Gait cluster, Gait phases, Classification, Gyroscope, Accelerometer

## Abstract

**Background:**

Gait symptoms and balance impairment are characteristic indicators for the progression in Parkinson’s disease (PD). Current gait assessments mostly focus on straight strides with assumed constant velocity, while acceleration/deceleration and turning strides are often ignored. This is either due to the set up of typical clinical assessments or technical limitations in capture volume. Wearable inertial measurement units are a promising and unobtrusive technology to overcome these limitations. Other gait phases such as initiation, termination, transitioning (between straight walking and turning) and turning might be relevant as well for the evaluation of gait and balance impairments in PD.

**Method:**

In a cohort of 119 PD patients, we applied unsupervised algorithms to find different gait clusters which potentially include the clinically relevant information from distinct gait phases in the standardized 4x10 m gait test. To clinically validate our approach, we determined the discriminative power in each gait cluster to classify between impaired and unimpaired PD patients and compared it to baseline (analyzing all straight strides).

**Results:**

As a main result, analyzing only one of the gait clusters constant, non-constant or turning led in each case to a better classification performance in comparison to the baseline (increase of area under the curve (AUC) up to 19% relative to baseline). Furthermore, gait parameters (for turning, constant and non-constant gait) that best predict motor impairment in PD were identified.

**Conclusions:**

We conclude that a more detailed analysis in terms of different gait clusters of standardized gait tests such as the 4x10 m walk may give more insights about the clinically relevant motor impairment in PD patients.

## Background

Parkinson’s disease (PD) is a degenerative disorder of the central nervous system which often affects the motor system [1]. Gait symptoms and balance impairment are major motor symptoms in PD patients [2]. These symptoms are associated with falls and freezing of gait (FOG) in PD which both have substantial impact on the patient’s quality of life [3]. Gait disorders and balance impairments in PD are most commonly characterized by clinical experts using the Unified Parkinson Disease Rating Scale part III (UPDRS-III) subitems “gait” and “postural stability” [4].

Clinical gait tests, often only focus on the middle part of a gait bout to avoid “confounding” in gait parameters by acceleration/deceleration [5] and hence might exclude potential clinically relevant gait phases. Also in technical gait assessments often only straight strides with assumed constant-velocity are considered, while acceleration/deceleration and turning strides are ignored, due to limitations in capture volume of commonly used systems like instrumented mats and optical Motion Capture. To overcome the technical limitations mentioned, a promising technology for objective and mobile gait analysis are inertial measurement units (IMUs), since they are relatively inexpensive and easy to integrate (e.g. in smart shoes [6]) compared to the before mentioned systems. Furthermore they offer the potential for home monitoring. Extensive research has been conducted in the field of sensor-based gait analysis including IMUs and other unobtrusive sensor technologies such as pressure insoles to enable mobile gait assessment for better disease monitoring [6–10].

Specifically, in home monitoring environments, the ratio of straight, constant-velocity gait to other gait phases (e.g. initiation, termination and turning) might decrease. This could be due to walking inside rooms or from one room to another typically requires turns and rarely allows more than a few straight strides.

Clinical assessment of short walking bouts during standard examinations by experienced movement disorder specialists includes all aspects of gait: gait initiation, acceleration, constant speed at different paces, deceleration, turning and termination. However, the evaluation is subjective and descriptive and lacks any standardization and comparability between raters. Especially, clinicians describe impairments of different gait phases as, e.g. slow gait (constant phase), prolonged initiation phase, pusher who cannot stop, postural instability with increased stride-to-stride variance of the constant and potentially also of the other gait phases. The clinical descriptive gait analysis is not represented by sensor-based gait assessment as defined so far [7–12]. Objective evaluation strategies e.g. gait speed or distance using stop watches typically only include highly defined phases of the selected gait test. Typically, the middle part of a short gait bout with assumed constant speed is evaluated. Wearable sensor technology such as inertial sensors allow to monitor each gait phase during standardized 4x10 m walks, however, only straight strides are typically included for gait parameter computing [7, 11, 12]. Thus, from a technical perspective, even though turning strides are excluded, still the first and last strides from a gait bout might increase the variance of the constant part (e.g. to define gait speed or stride length). From a clinical perspective, especially the acceleration and deceleration phase of gait bouts might be of even more relevance for certain aspects of gait impairment (e.g. initiation, balance and postural control).

In the following, gait and turning phases are defined:

Gait initiation is defined as the transition from motionless standing to steady-state gait and characterized by anticipatory postural adjustment where the center of pressure is shifted [13, 14]. In PD it is well-known that particularly problems with the initiation of gait can often cause FOG [14, 15].

Gait termination is the transition from steady-state gait to still standing after deceleration [16]. There exists very little research studying gait termination in PD patients [16]. Bishop et al. [17] found that PD patients have more difficulties to generate sufficient braking impulse during unplanned gait terminations.

Gait transitioning in the present study is defined as the transition from straight walking to 180^∘^ turns and vice versa. These gait phases require the ability to decelerate and accelerate before and after turning 180^∘^, which demands complex adjustments in gait and posture. Mak et al. [18] showed that PD patients have difficulties switching from straight walking to turning. Mariani et al. [19] developed an algorithm to detect transition strides and reported that PD patients took significantly more time for gait transitioning during a timed up and go (TUG) test. Schlachetzki et al. [11] stated that PD patients have problems with walk to turn transitions due to disease-characteristic gait impairment and deficits in postural balance control.

Turning (changing directions) while walking is an important component of mobility and requires control of dynamic balance. During daily live activities 50% of strides taken are turning strides [20]. Mancini et al. [21] showed that turning can be a good indicator for FOG in PD. There are several studies showing that PD patients have difficulties in turning and that it might be a good indicator for risk of falling [20–25].

Current research results in inertial sensor-based gait analysis for PD patients focus on the evaluation of all detected straight strides [7, 11, 12] which could bias the resulting mean and coefficient of variance (CV) of stride parameters by mixing steady state walking with non-steady state walking strides. Hence the analysis and comparison of clinically relevant information in different phases of walking (constant, initiation, termination, transitioning and turning) are of great interest.

The aim of this study was to define gait clusters that have the potential to obtain the distinct gait parameters from the different gait phases within a standardized 4x10 m gait test (Fig. 2) performed at the movement disorder unit (Point-of-Care visit) into gait clusters. These gait clusters were defined by novel, unsupervised methods as: baseline (including all straight strides), constant gait (straight strides excluding acceleration and deceleration strides), non-constant gait (which includes gait initiation, termination and transitioning) and turning (includes all turning strides).

We hypothesize that different gait phases clustered by different mathematical approaches provide more clinically relevant information than the complete 10 m gait bout, and that an automated clustering approach can isolate this information.

To clinically validate this approach, we compared the discriminative power in each gait cluster to classify between impaired and unimpaired PD patients in terms of the established UPDRS-III subitems “gait” and “postural stability” [4]. As an evaluation measure commonly used in medical decision making we calculated the area under the curve (AUC) based on the receiver operating characteristic (ROC) curve analysis [26, 27].

## Materials and methods

**Fig. 1:**
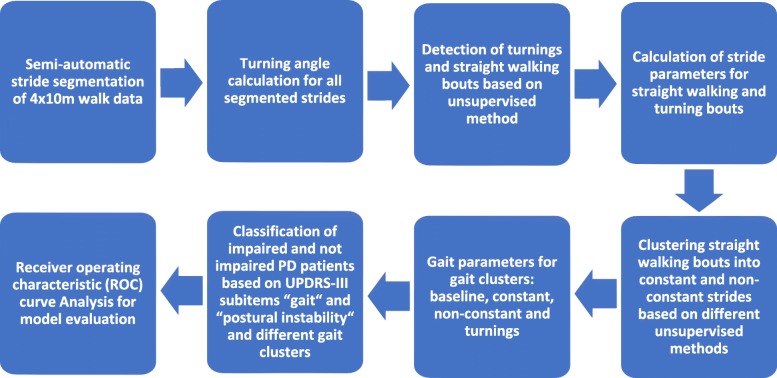
Flowchart illustrating the different steps applied to compare the clinically relevant information about the motor impairment in PD patients in different gait clusters

The overall approach used in this study is illustrated in Fig. 1. The individual steps will be explained in the following sections.

**Fig. 2:**
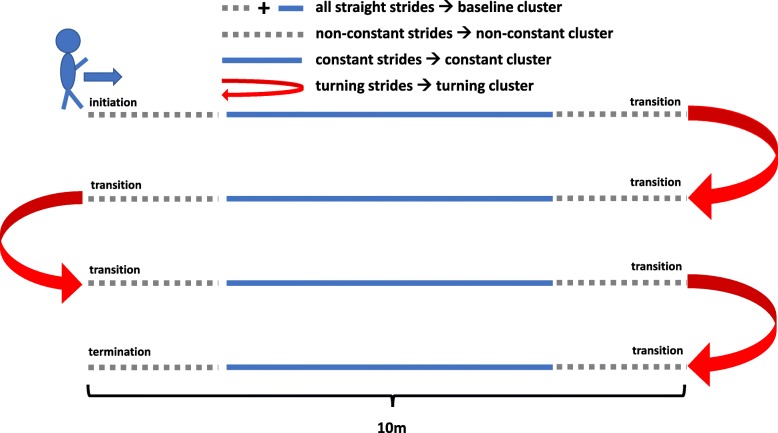
Model of transformation of clinically relevant gait phases into gait clusters within the 4x10 m test. The turning cluster (red) was determined based on the algorithm described in Turning isolation. A set of parameters per subject was calculated for each of the defined gait clusters. The constant (blue) and non-constant (gray) gait clusters were defined based on the corresponding clustering methods described in Clustering straight walking bouts into constant and non-constant gait cluster. The baseline cluster consists of all straight strides and is thus the combination of all constant (blue) and non-constant (gray) strides

### Database

Parkinson’s disease patients (n=119) were selected from a larger stratified patient cohort visiting the Movement Disorders Outpatient Clinic of the Department of Molecular Neurology at the University Hospital Erlangen, Germany. PD patients were selected based on two criteria: 1) all required data (gait and clinical) for the analysis were complete and 2) to balance the sample size between PD patients with presence (impaired) and absence (unimpaired) of motor impairments in terms of the UPDRS-III subitems “gait” and “postural stability”. The study was approved by the local ethics committee (IRB-approval-Re. -No. 4208, 21.04.2010, IRB, Medical Faculty, Friedrich-Alexander University Erlangen-Nuernberg, Germany) and all participants gave written informed consent according to the Declaration of Helsinki.

Sporadic PD was defined according to the Guidelines of the German Association for Neurology (DGN), which are similar to the UK PD Society Brain Bank criteria [28]. Inclusion criteria consisted of PD patients with Hoehn and Yahr disease stage (H&Y) between I and III, and the ability to walk independently without a walking aid. PD patients were excluded if other neurological disorders, severe cardiovascular or orthopedic conditions affected the assessments.

All PD patients were clinically (UPDRS-III [4]) and biomechanically (gait analysis) investigated in stable ON medication without presence of clinically relevant motor-fluctuations during the assessments.

For the UPDRS-III subitem “postural stability”, the physician is instructed to invoke sudden displacements of the patient by pulling his/her shoulders backwards and scoring the patients response on a five level scale: 0 (normal recovery) – 1 (retropulsion with unaided recovery) – 2 (would fall if unaided) – 3 (tendency to spontaneous imbalance) – 4 (requires assistance to stand) [4].

For the UPDRS-III subitem “gait”, the patient is instructed to walk at least 10 m away from and torwards the examiner. The patients gait is then evaluated on a five level scale: 0 (normal) – 1 (walks slowly, may shuffle with short steps, no festination or propulsion) – 2 (walks with difficulty, little or no assistance, some festination, short steps or propulsion) – 3 (severe disturbance, frequent assistance) – 4 (cannot walk) [4].

For IMU-based gait assessment, all participants walked freely at a comfortable, self-chosen speed in an obstacle-free and flat environment for 4x10 m. After each 10 m walk, participants were instructed to turn 180^∘^ [11]. Figure 2 illustrates the 4x10 m gait test including the gait clusters defined for this work.

In order to clinically validate our clustering approaches, we grouped PD patients based on the UPDRS subitems “gait” and “postural stability” into the classes “impaired” if the corresponding clinical score is nonzero (> 0) and to the class “unimpaired” otherwise (= 0). These two subitems were chosen since they are the most reflective ones for PD gait. The distribution and characteristics of PD patients analyzed in this study are shown in Table 1.

**Table 1:** Distribution and characteristics of PD patient cohort

	“gait”	
	unimpaired (n=51)	impaired (n=50/18)
	(“gait” = 0)	(“gait” = 1/2)
Age (years, mean ± std)	58.47 ± 12.42	60.23 ± 10.83
Height (cm, mean ± std)	172.27 ± 9.08	172.99 ± 9.36
Weight (kg, mean ± std)	76.73 ± 14	78.68 ± 14.37
	“postural stability”	
	unimpaired (n=58)	impaired (n=45/16)
	(“postural stability” = 0)	(“postural stability” = 1/2)
Age (years, mean ± std)	57.22 ± 11.06	59.23 ± 11.32
Height (cm, mean ± std)	173.07 ± 9.44	172.31 ± 9.05
Weight (kg, mean ± std)	78.47 ± 14.88	77.25 ± 14.31

### Measurement system

**Fig. 3:**
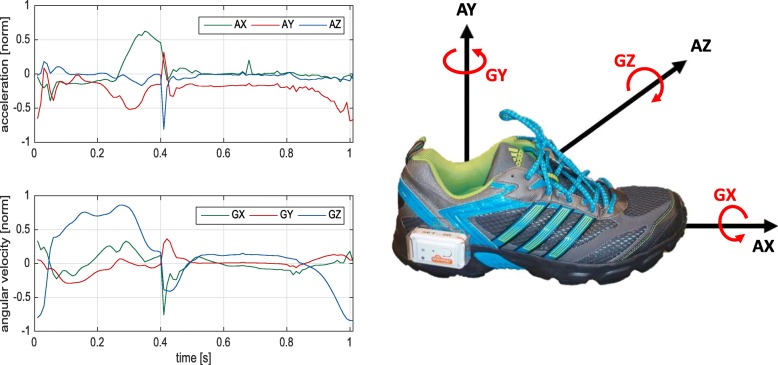
(**Left**) Accelerometer and gyroscope data (normalized by range) for one exemplary stride; (**Right**) Sensor (Shimmer 2R) placement on the lateral side of each shoe and axes definition (AX, AY and AZ form three dimensions of accelerometer and GX, GY and GZ form three dimensions of gyroscope)

Gait data were recorded with an inertial measurement system consisting of two Shimmer 2R/3 inertial measurement units (Shimmer Sensing, Dublin, Ireland [29]) [11, 12]. Sensors were attached to the lateral side of each shoe (Fig. 3). Each sensor consists of a three-axis accelerometer (range (Shimmer 2R (3)): ± 6 g (± 8 g)) and a three-axis gyroscope (range (Shimmer 2R (3)): ± 500^∘^/ sec (± 1000^∘^/ sec)). Data were recorded with a sampling rate of 102.4 Hz.

The x-axis was defined in posterior–anterior direction, y-axis was in superior–inferior direction, and z-axis was in medio-lateral direction. Figure 3 illustrates the measurement system with axis definitions for the accelerometer and gyroscope and an example signal of an exemplary stride.

### Semiautomatic stride segmentation

To ensure that all strides (including turning and transition strides) were captured, semiautomatic stride segmentation was applied, since the goal of this study was to compare the information decoded in different gait clusters.

**Fig. 4:**
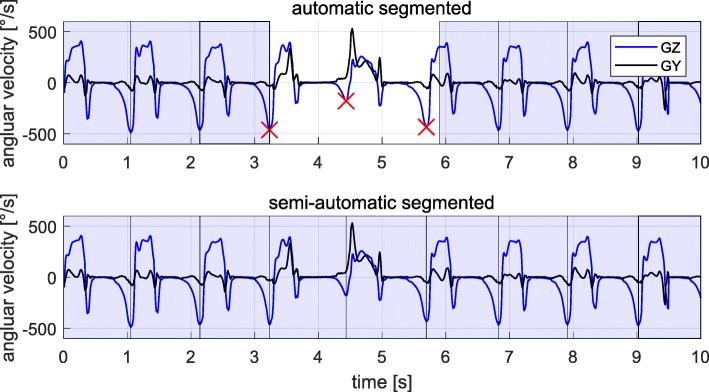
Semi-automatic stride segmentation of a sequence of a 4x10 m gait test signal. (**Top**) Strides automatically segmented by the DTW algorithm using only the GZ axis as a template (blue boxes). The minima in the GZ signal (red crosses) were used to define stride borders for missing or wrongly segmented strides. (**Bottom**) Fully segmented sequence of a 4x10 m gait test signal. Missing or wrongly segmented strides were manually added or corrected based on the minima in the GZ signal

At first the Dynamic Time Warping (DTW) algorithm presented in [30] only using the GZ axis was applied to initially detect as many strides as possible. DTW is a template-based approach to segment single strides within a gait sequence [30]. After the automatic stride detection using DTW, wrongly (e.g. the stride boundaries are not aligned with the minima of the GZ signal) segmented strides were corrected and missing strides were added manually based on minima in the GZ signal. Figure 4 shows an example for a semi-automatically annotated sequence of a 4x10 m gait test including straight and turning strides.

### Turning isolation

In the case of a 4x10 m walking test we anticipated three turning sequences which were to be isolated from the four straight walking bouts. Therefore, the following unsupervised algorithm was applied to each foot separately.

**Fig. 5:**
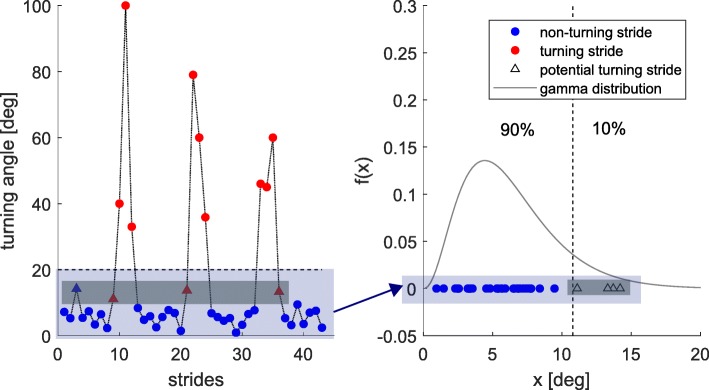
Turning isolation algorithm for an exemplary 4x10 m gait sequence and one foot. (**Left**) Absolute value of the TA including: Strides with TA larger than 20^∘^ (red circles), straight strides (blue circles), potential turning strides adjacent to already assigned turning strides (red triangles) and potential turning strides not adjacent to already assigned turning strides (blue triangles). (**Right**) Gamma distribution fitted based on all strides with TA smaller than 20^∘^. The largest 10% are potential turning strides (triangles) and the remaining strides are straight strides (blue circles)

First of all, turning angles (TA) for each segmented stride were calculated based on the algorithm described in [19] from mid stance (MS) to MS. All strides with an absolute TA larger than 20^∘^ were then classified as turning strides. The threshold was chosen based on a study of Mariani et al. [31]. For all remaining strides, a gamma distribution was fitted based on the absolute value of the TA and the upper 10% TA were classified as turning strides if they were adjacent to one of the already assigned ones. A gamma distribution was chosen because we only considered positive values (absolute value of TA) and expected a single-tailed (large absolute values of TA) distribution.The general approach is shown in Fig. 5 for an exemplary 4x10 m gait sequence and one foot.

### Clustering straight walking bouts into constant and non-constant gait cluster

After isolation of the turning phases from the 4x10 m gait test sequence, the four straight 10 m walking bouts were further analyzed. The goal was to cluster straight strides into constant and non-constant gait (initiation, termination and transitioning) for each 10 m walking bout separately. Detected phases of gait initiation, termination and transitioning were summarized as the non-constant gait cluster and hence the remaining part of a 10 m bout as the constant gait cluster as illustrated in Fig. 2.

**Fig. 6:**
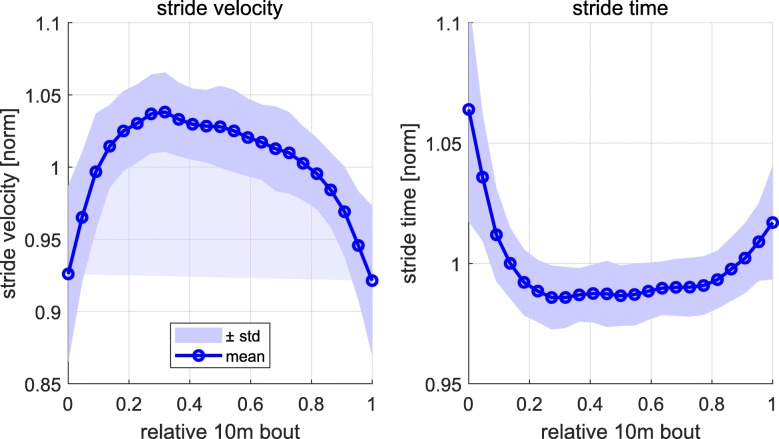
Stride velocity (left) and stride time (right) over a normalized 10 m bout. The mean and standard deviation was calculated over all subjects. For each subject the mean over all four 10 m bouts was computed, while each parameter was normalized by the mean value over each 10 m bout

We used stride velocity and stride time as parameters to cluster straight walking strides into constant and non-constant gait clusters. The stride time was calculated based on the time difference of two consecutive heel strike events as described in [32]. The stride velocity is the ratio of stride length and stride time. Stride length was computed through a double integration of the gravity corrected accelerometer signal between two consecutive MS events as described in [32]. It is reasonable to assume that subjects will accelerate at the beginning and decelerate at the end of each 10 m walking bout. Figure 6 shows the mean ± standard deviation of the stride velocity and stride time over all subjects and all 10 m bouts. The parameters for each subject were normalized by the mean value over each 10 m bout. The stride velocity was normalized by the body height of the patients in order to control for height-dependent gait characteristics [33]. To account for different numbers of strides per subject for a 10 m bout linear interpolation was applied over all 10 m bouts of one subject to compute the mean over the four 10 m bouts. Finally, linear interpolation was applied over all subjects and the 10 m bout has been normalized between 0 and 1.

Several unsupervised methods to cluster straight walking bouts into constant and non-constant strides were developed and applied.

The first method and simple approach was to assign the first and last *N%*,*N*∈{5,10,15,20,25} of strides of each 10 m bout to the non-constant gait cluster and the remaining to the constant gait cluster respectively. We will refer to this method as *N%crop*.

More selective approaches were developed and implemented to assign strides to the non-constant gait cluster for each 10 m bout. Therefore we defined a lower and an upper threshold for either the stride velocity or stride time within which we assigned a stride as constant and outside as non-constant. These two thresholds were determined based on four different methods.

The first method was based on the median of the stride velocity or stride time for each 10 m bout. The upper and lower threshold were defined as 5% below and above this value after normalization with the median. We will refer to this method as *median vel/t*.

The second approach was to fit a normal distribution to the stride velocity or stride time for each 10 m bout and define the upper and lower threshold as the mean ± the standard deviation. We will refer to this method as *distr vel/t*.

The third method was based on the K-means clustering method [34] with five random initiations of the mean. The result within these five trials with the biggest separation of clusters was chosen as the final result. The parameter K was set to 3 because we anticipated for *K*=3 that there will be a mean for slow, medium and fast strides during a 10 m bout defined by the algorithm. The means for the slower and faster strides were then used as the lower and upper thresholds. We will refer to this method as *3means vel/t*.

The fourth and last method was based on percentiles. The lower threshold was defined as the 25% and the upper as the 75% percentile of the stride velocity or stride time within each 10 m bout. We will refer to this method as *perc vel/t*.

**Fig. 7:**
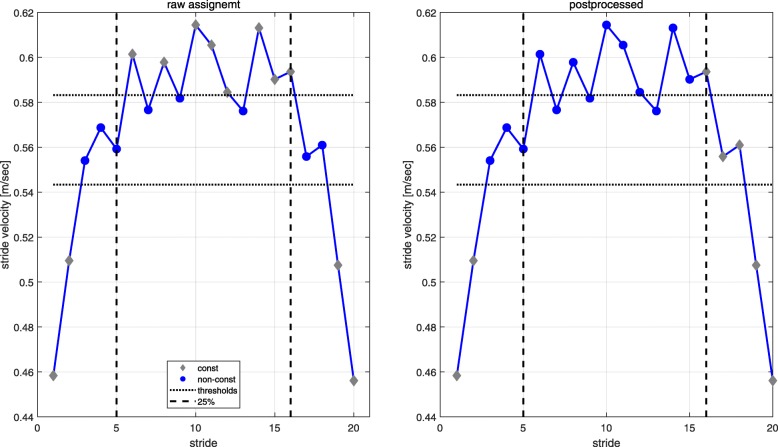
Illustration of the algorithm for clustering an exemplary 10 m bout into constant and non-constant gait clusters. The example is based on the method *distribution vel* and shows the raw (left) and the postprocessed (right) cluster assignment

After all strides were classified as constant or non-constant a post processing algorithm was applied to ensure the order of gait acceleration, steady state gait and gait declaration as consecutive periods of each 10 m walking bout. We assumed that gait acceleration and deceleration can only take place within the first and last 25% of each 10 m walking bout. Within the first 25% we assigned all strides until the last one assigned as non-constant based on the thresholds as gait acceleration. The same procedure was applied to assign strides as gait deceleration for the last 25% looking from the end to the beginning of each 10 m bout. All remaining strides between the last acceleration and first deceleration stride within the 25% bands were then assigned to the constant gait cluster. The acceleration and deceleration strides were then both assigned to the non-constant gait cluster for further analysis. The 25% thresholds for the post processing were chosen based on the work of Miller et al. [35] stating that three strides are necessary to reach constant-velocity gait. Perry et al. [36] mentioned that at least 3 m are needed at the end to measure enough constant strides in the middle part of a 10 m walk bout. The algorithm with raw and post processed gait cluster assignment is illustrated in Fig. 7.

### Calculation of gait parameters

The following spatio-temporal parameters were calculated for all straight strides: stride time, swing time, stance time, stride length, stride velocity, heel strike angle, toe off angle, maximal toe clearance, path length and turning angle [19, 32, 37, 38]. Path length and stride length were like stride velocity, also normalized by the body height of the patients in order to control for height-dependent gait characteristics [33]. The swing and stance time were both given relative to the stride time. For each subject we calculated the mean and CV over all constant and non-constant strides (assigned based on algorithm described in Clustering straight walking bouts into constant and non-constant gait cluster section) from a 4x10 m gait test resulting in one mean and one CV value per parameter for each subject and gait cluster.

Parameters calculated per turn were: number of strides and turning time. For each subject we calculated the mean of each of the two parameters over all three turns. Furthermore we calculated the following parameters per turning stride for all turns: stride length, stride time, stride velocity and path length [19, 32]. We then again computed the mean and CV over all turning strides per subject over all turns.

We finally had a set of parameters per subject for each of the defined gait clusters: *baseline*, *constant*, *non-constant* and *turning*. The defined gait clusters in terms of the 4x10 m gait test are illustrated in Fig. 2.

### Assessing clinical information in each cluster

In order to compare the discriminative power in each gait cluster we applied a state of the art machine learning methodology pipeline for each gait cluster and clustering method. This pipeline was applied for binary classification to distinguish between impaired and unimpaired PD patients based on the UPDRS-III subitems “gait” and “postural stability”. Note that the clustering methods described above are independent of the class labels and hence can be seen as a preprocessing step.

#### Evaluation procedure

Patients were assigned to the class *impaired gait/posture* if the corresponding clinical score is nonzero (> 0) and to the class *unimpaired gait/posture* otherwise (= 0). The complete data set was randomly partitioned into 60% for training and 40% for testing. Furthermore, the training data set was then balanced based on both groups impaired and unimpaired [27] and Table 2 shows the resulting distribution of samples for the “gait” and item “postural stability”.

**Table 2:** Distribution of training and test data used for classification

Training set		Test set	
unimpaired	impaired	unimpaired	impaired
UPDRS-III item “gait”			
(item “gait” = 0)	(item “gait” = 1/2)	(item “gait” = 0)	(item “gait” = 1/2)
n=31	n =20/11	n=20	n=20/7
UPDRS-III item “postural stability” (“pi”)			
(item “pi” = 0)	(item “pi” = 1/2)	(item “pi” = 0)	(item “pi” = 1/2)
n=35	n =20/15	n=23	n=18/6

We applied a receiver operator characteristics (ROC) analysis and calculated the area under the curve (AUC) as our primary evaluation measure [26, 27]. The major advantage of this measure is that it evaluates the classification performance for different configurations of a classifier and is as such more general then e.g. the classification accuracy. Therefore, these evaluation measures are a common choice in the machine learning community and for medical decision making [27].

For each of the defined gait clusters, gait parameters were extracted as already outlined in “Calculation of gait parameters” section. For each classification task the top five parameters were selected based on the fisher score on the training set [39]. The fisher score is a measure for the discriminative power of a parameter and is calculated as the squared difference of class means weighted by the sum of the class variances.

We applied a support vector machine (SVM) algorithm as a classification algorithm with linear and radial basis function (RBF) kernel [40]. Boosting and bagging algorithms were applied in preliminary experiments but did perform inferior in comparison to SVM. The SVM parameters C and *γ* were found in the inner 5-fold cross validation with a grid search over *C*={0.01,0.05,0.1,0.5,1,5,10,20,30,40,50,100} and *γ*={0,0.01,0.05,0.1,0.5,1,5,10,20,40,50,100}. Note that a selection of *γ*=0 would coincide with selection of a linear kernel while other choices result in an RBF kernel. The optimal model parameters were selected based on the maximum AUC and then used to train a model on the whole training set.

We finally evaluated the optimal classification model selected on the unseen test data. The AUC based on the ROC curve [26] was then calculated for the evaluation.

## Results

**Fig. 8:**
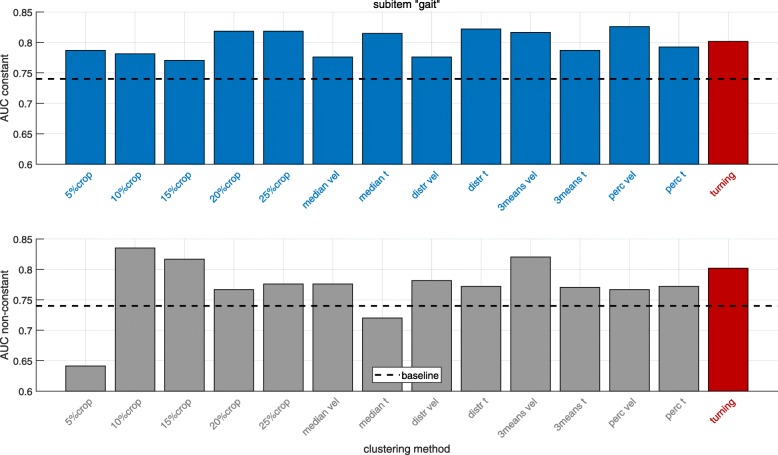
Classification results for distinguishing impaired and unimpaired PD patients based on the UPDRS-III item “gait” for the constant (top) and non-constant (bottom) gait clusters. The dashed line indicates the corresponding baseline result including all straight strides. “turning” is based on parameters extracted from the turning cluster. The methods are corresponding to the definitions in “Clustering straight walking bouts into constant and non-constant gait cluster” section

**Fig. 9:**
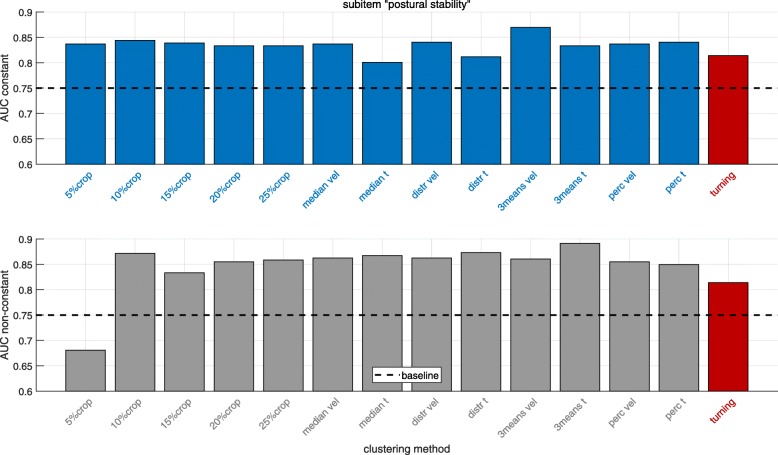
Classification results for distinguishing impaired and unimpaired PD patients based on the UPDRS-III item “postural stability” for the constant (top) and non-constant (bottom) gait clusters. The dashed line indicates the corresponding baseline result including all straight strides. “turning” is based on parameters extracted from the turning cluster. The methods are corresponding to the definitions in “Clustering straight walking bouts into constant and non-constant gait cluster” section

Classification results (AUC values) distinguishing impaired and unimpaired PD patients based on the UPDRS-III subitems “gait” and “postural stability” were calculated for different gait clusters and clustering methods as the clinical validation of the clustering approaches. In Figs. 8 and 9 results are presented for the UPDRS-III subitems “gait” and “postural stability” respectively comparing different, previously defined gait clusters. Since in inertial sensor-based gait analysis often all straight strides are included when computing gait parameters (baseline), the results highlight the comparison of AUC values derived from different gait clusters, which potentially decode the different gait phases, to baseline for the two analyzed UPDRS-III subitems.

For the UPDRS-III subitem “gait” (Fig. 8) the AUC value for the baseline cluster is 0.74. All clustering methods yielding to a better classification for the gait cluster “constant” compared to baseline. The AUC value increases up to around 11% (AUC = 0.82) relative to baseline for multiple clustering methods (20*%crop*,25*%crop*, *median t*, *distr t* and *3means vel* and *perc vel*).

In the gait cluster “non-constant” the AUC values increase for all clustering methods (except 5*%crop* and *median t*) up to 13% (AUC = 0.84) (clustering method 10*%crop*). For the turning cluster there is an increase of 8% (AUC = 0.80) compared to baseline.

Looking at the results for the UPDRS-III subitem “postural stability” (Fig. 9), the baseline AUC value is 0.75. Again, the gait cluster “constant” gives for all clustering methods a better classification performance compared to baseline. The AUC value increases up to 12% (AUC = 0.84) for several clustering methods and up to 16% (AUC = 0.87) for the *3means vel* method.

Considering the gait cluster “non-constant”, the classification improves for all methods except 5*%crop*. The AUC increases up to 19% (AUC = 0.89) (clustering method *median t*). For the turning cluster there is an increase of 8% (AUC = 0.81) compared to baseline.

To sum up, for both UPDRS-III subitems, each of the defined gait clusters yields a higher AUC value compared to analyzing all straight strides (baseline).

Different gait parameters were selected based on the methodology described in “Assessing clinical information in each cluster” section. Table 3 shows the parameters selected for the UPDRS-III subitems “gait” and “postural stability” and different gait clusters. Note that for the constant and non-constant gait clusters more than 5 parameters could be reported since different clustering methods might lead to a different set of selected parameters.

**Table 3:** All selected parameters for each gait cluster and UPDRS-III subitem

Gait cluster	UPDRS-III item “gait”	UPDRS-III item “postural stability”
baseline	toe off angle (mean),	stride velocity (CV),
	stride velocity (CV),	stride velocity (mean),
	heel strike angle (CV),	path length (mean),
	stride velocity (mean),	stride time (CV),
	swing time (CV)	swing time (CV)
constant	toe off angle (mean),	stride time (CV),
	stride velocity (mean),	stride velocity (mean),
	stride time (CV),	path length (mean),
	path length (mean),	stride velocity (CV),
	heel strike angle (CV),	toe off angle (mean),
	toe off angle (CV)	stride length (mean)
non-constant	toe off angle (mean),	stride velocity (CV),
	stride velocity (mean),	stride velocity (mean),
	heel strike angle (CV),	path length (mean),
	path length (mean),	stride length (mean),
	stride length (mean)	toe off angle (mean)
turning	path length (mean),	stride length (mean),
	stride length (mean),	number of strides,
	stride velocity (mean),	path length (mean),
	number of strides,	stride velocity (mean),
	turning time	turning time

Note that for the gait clusters constant and non-constant more than 5 parameters could be listed since different clustering methods might lead to different parameter choices

## Discussion

The aim of this study was to define gait clusters, which potentially include the clinically relevant information from distinct gait phases in the standardized 4x10 m gait test. We hypothesized that the clustering approaches could isolate the information from different gait phases and hence provide more clinically relevant information than the complete 10 m bout (baseline). To clinically validate this approach, we compared the discriminative power (classification of impaired and unimpaired PD patients based on UPDRS-III subitems “gait” and “postural stability”) in the mathematically defined gait clusters compared to analyzing all strides.

The results for the constant gait cluster (top plots in Figs. 8 and 9) show that all clustering methods described in Clustering straight walking bouts into constant and non-constant gait cluster were giving better classification results than baseline for both the UPDRS-III subitems “gait” and “postural stability”. This confirms the hypothesis that clustering constant strides improves the classification of impaired and unimpaired PD patients compared to baseline (analyzing all straight strides). A possible explanation for this result might be that combining all straight strides includes gait initiation, termination and transitioning could bias the calculated mean and CV parameters. Using constant strides, the clustering methods 20*%crop*,25*%crop*, median t, distr t, 3means vel and *perc vel* were giving (in terms of the UPDRS-III item “gait”) substantially larger AUC’s (all AUC = 0.82) compared to the baseline cluster (AUC = 0.74). For the UPDRS-III item “postural stability” the method *3means vel* gave a substantially higher AUC value (AUC = 0.87) in comparison to baseline (AUC = 0.75). Other methods like e.g. *N%crop*, median vel, dist vel also showing superior results (AUC = 0.83-0.84) compared to baseline (AUC = 0.75) as well.

Interestingly, almost all clustering methods except the 5*%crop* and *mediant* (gait) approach were giving superior results for the non-constant gait cluster in comparison to baseline for the UPDRS-III subitems “gait” and “postural stability” as presented in Figs. 8 and 9 on the bottom. This confirms the hypothesis that non-constant gait cluster yields a better classification of impaired and unimpaired PD patients compared to all straight strides. Higher AUC values for the non-constant cluster in the item “postural stability” indicate that balance deficits are more reflected by gait alterations in gait initiation, acceleration and deceleration than differences in straight walking [14, 15].

The overall clinical gait assessment rather focuses on quantitative gait characteristics as stride length and gait velocity, which is mostly reflected in the constant gait cluster. All constant clusters show superior associations to clinically relevant gait symptoms compared to baseline. This indicates that parameters generated from initiation, acceleration and deceleration strides negatively influence the evaluation of gait symptoms in PD. More similar results in comparison to the clinician’s ratings are presented by constant strides. This could be explained by a) that the computed mean and CV parameters are biased in the baseline cluster as mentioned above and b) that gait initiation, termination and transitioning are especially impaired in PD patients [14–18, 41] which is decoded in the non-constant gait cluster. The method 5*%crop* was inferior to baseline because some patients may not take enough strides (less than 10) per 10 m bout leading to very few strides to be analyzed. Worth mentioning on the other hand is that only analyzing the first and last 10% of strides in each 10 m bout gave even better or similar results compared to the constant gait cluster and hence baseline for the UPDRS-III subitems “gait” and “postural stability” (AUC = 0.84 and AUC = 0.87).

Another interesting observation is that the simple *N%crop* methods were performing similarly well in comparison to the more selective clustering approaches for the constant and non-constant gait clusters and both UPDRS-III items. Hence there was best clustering method identified. Since the major goal of this study was to compare the classification performance in each gait cluster compared to analyzing all straight strides.

The turning cluster led also for both the UPDRS-III subitems “gait” and “postural stability” (AUC = 0.80 and AUC = 0.81) to superior results compared to baseline (AUC = 0.74 and AUC = 0.75), which confirms the hypothesis that the turning cluster yields better classification of impaired and unimpaired PD patients compared to baseline. This also coincides with results from several studies showing that PD patients have severe problems performing turning tasks [20–25].

In Table 3 the selected parameters are listed for the classification tasks. Slow gait, small steps and shuffling are reflecting bradykinesia in PD patients. Schlachetzki et al. [11] pointed out that for PD patients typically slow gait is reflected by stride velocity, small steps by the stride length (and hence path length) and shuffling by toe off and heel strike angles. The selected parameters were reported to indicate disease staging in PD in [11]. Parameters selected for the turning cluster are confirming that PD patients with motor impairment have more difficulties with turnings reflected in more time taken, more, smaller and slower steps [11, 20–25].

One limitation of this work is that semi-automatic segmentation of strides was applied, especially to include turning and transition strides. Hence this approach cannot be fully automated and requires some manual work for labeling.

Another limitation is that the method for turning isolation is not validated since the definition of turning is still an open research topic. The same holds for the definition of constant and non-constant strides. In this study, we focused on the clinical validation of the clustering methods. The clinical validation has the limitation that scores of 1 and 2 were merged together into one group. This was done as a proof of concept of the clustering approaches in this study. By analyzing the UPDRS-III subitems “gait” and “postural stability” it is not possible to distinguish between different domains of balance impairments.

Furthermore, only data from the 4x10 m test were considered for this study. The limited number of strides within a 4x10 m gait test led to the approach to combine gait initiation, termination and transitioning into one gait cluster, namely non-constant as illustrated in Fig. 2. Due to the limited amount of consecutive straight strides the CV parameters should be interpreted with caution. In the literature, different values for the number of strides needed for robust measure of gait variability ranging from at least 15 to 300 are reported [42]. For the non-constant gait cluster, the CV is calculated from fewer strides and thus more sensitive to “outliers”. Therefore, the CV might contain the clinically relevant information, meaning that “abnormal stride” - especially in the non-constant cluster are highly relevant in the sense that they contain clinically relevant information leading to “clinical decision support”.

## Conclusion and future work

A novel set of methods was developed and applied to identify different gait clusters (constant, non-constant and turning) within the standardized 4x10 m gait test. Classification experiments with a cohort of 119 PD patients were performed to compare the clinically relevant information (presence and absence of motor impairments in terms of the UPDRS-III subitems “gait” and “postural stability”) in the defined gait clusters to baseline (analyzing all straight strides). The results of this study suggest that: 1) isolation of constant gait or conversely exclusion of non-constant gait (initiation, termination, transitioning) from a straight walking bout, 2) only analyzing non-constant strides and 3) only analyzing turning strides separately, could include superior clinical information to distinguish impaired from unimpaired PD patients compared to analyzing all straight strides in the standardized 4x10 m gait test. The information extracted from gait initiation, termination, transition and turnings have the potential to give important insights about the impairment of gait and postural stability in PD patients. These findings are especially interesting for home monitoring applications where the ratio of straight, steady state walking to more dynamic motor tasks such as turning, gait initiation, gait termination and transitioning decreases due to the nature of available walking paths. We recommend clustering constant gait when analyzing straight strides. Furthermore it is worth analyzing non-constant strides and turning strides separately if possible.

Our analysis suggests that the most relevant parameters for straight walking (baseline, constant and non-constant gait clusters) to distinguish between impaired and unimpaired PD patients seem to be stride velocity, stride and path length, stride time and swing time, as well as toe off and heel strike angle. For the turning cluster we would recommend focusing on the number of strides and turning time in addition to stride and path length and stride velocity. It would be interesting to investigate further parameters derived from the defined gait clusters to see if they improve the classification. For example, could an additional sensor at the posterior trunk at L5 potentially improve the turning detection algorithm and provide further parameters associated to trunk movements and center of mass [43] to eventually improve classification. Since it is possible to calculate stride times with a sensor positioned at L5 as described in [43], the stride time-based clustering methods (see “Clustering straight walking bouts into constant and non-constant gait cluster” section) could potentially be applied for such sensor setup as well.

Future work and applications could include the development of a stride segmentation algorithm which can detect and segment strides from all gait clusters defined in this study automatically.

The analysis of different gait clusters in e.g. the 2 min walk test [44] which includes more and longer straight walking bouts might be interesting to further confirm the findings of this study. The larger amount of strides in a 2 min test enables more force of expression when dividing strides in the non-constant gait clusters. Finally, it would be interesting to investigate how the findings of this study could be transferred to supervised and non-supervised at-home data.

## Data Availability

The datasets used and/or analyzed during the current study are available from the corresponding author on reasonable request and after approval by the ethical committee in case patient related data is requested.

## Abbreviations

AUC: Area under the curve
CV: Coefficient of variance
DTW: Dynamic time warping
FOG: Freezing of gait
IMU: Inertial measurement unit
MS: Mid stance
PD: Parkinson’s disease
RBF: Radial basis function
ROC: Receiver operating characteristic
SVM: Support vector machine
TA: Turning angle
TUG: Timed up and go
UPDRS - III: Unified parkinson disease rating scale part III
