# Correction to: Development and clinical validation of inertial sensor-based gait-clustering methods in Parkinson’s disease

**DOI:** 10.1186/s12984-019-0567-z

**Published:** 2019-07-26

**Authors:** An Nguyen, Nils Roth, Nooshin Haji Ghassemi, Julius Hannink, Thomas Seel, Jochen Klucken, Heiko Gassner, Bjoern M. Eskofier

**Affiliations:** 1 Machine Learning and Data Analytics Lab, Department of Computer Science, Friedrich-Alexander-University Erlangen-Nürnberg (FAU), Carl-Thiersch-Straße, 2b, 91052 Erlangen, Germany; 2 Department of Molecular Neurology, University, Hospital Erlangen, Friedrich-Alexander University Erlangen-Nürnberg (FAU), Schwabachanlage 6, 91054 Erlangen, Germany; 3 Control Systems Group, Department of Electrical Engineering and Computer Science, Technische, Universität Berlin (TUB), Einsteinufer 17, 10587 Berlin, Germany

**Correction to: J Neuroeng Rehabil 2019;16:77**

**https://doi.org/10.1186/s12984-019-0548-2**

The original article [1] contained an error whereby Fig. 6 contained a minor shading glitch affecting its presentation. This has now been corrected.
